# Supplementary material for: Development and validation of the Self-Efficacy in Addressing Menstrual Needs Scale (SAMNS-26) in Bangladeshi schools: A measure of girls’ menstrual care confidence
Source: PLoS One. 2022 Oct 6;17(10):e0275736. doi: 10.1371/journal.pone.0275736 (PMC9536616; doi:10.1371/journal.pone.0275736)

S1 Fig. Integration of a self-efficacy sub-study to develop and validate the Self-Efficacy in Addressing Menstrual Needs Scale within the main study '*Piloting MHM interventions among urban and rural schools in Bangladesh*', 2017-2018

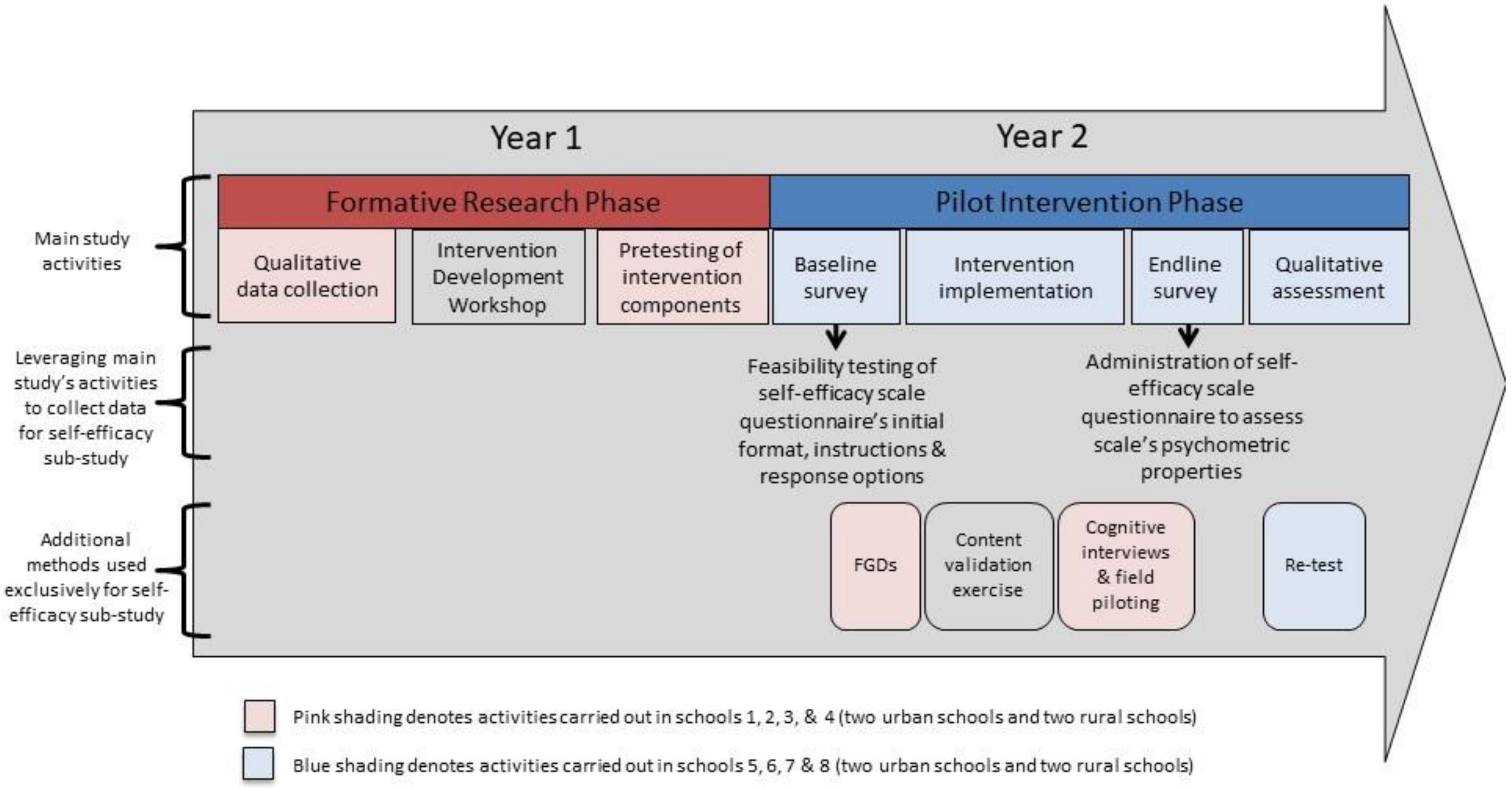

Supplement: S1 Fig — (PDF) [file pone.0275736.s001.pdf]
