# Supplementary material for: Development and validation of the Self-Efficacy in Addressing Menstrual Needs Scale (SAMNS-26) in Bangladeshi schools: A measure of girls’ menstrual care confidence
Source: PLoS One. 2022 Oct 6;17(10):e0275736. doi: 10.1371/journal.pone.0275736 (PMC9536616; doi:10.1371/journal.pone.0275736)
Supplement: S2 Fig — (PDF) [file pone.0275736.s002.pdf]

S2 Fig. Example vignette activity as part of focus group discussions with schoolgirls during the development of the Self-Efficacy in Addressing Menstrual Needs Scale in Bangladesh, 2017-2018

Purpose: To elicit a list of tasks required to address menstrual needs across multiple categories and identify their relative levels of difficulty

Introductory information: "*We are going to talk about a girl named Mim. Let's imagine that Mim is a girl your age that lives in this area.* **[Hold up illustration of Mim]** *I will begin telling you a little about Mim and the different issues she faces, and I want you to help me complete the stories or tell me what advice you would give to Mim in each situation. It may be helpful to think of Mim as one of your own friends. What would they do in each situation?"*

[Category 1: Obtaining menstrual materials]
Elicit task list: "*Mim has just started her period and learned that there are some ways to absorb the blood by using clean cloth or disposable pads, but she is unsure how to obtain these things. Can you tell her what she must do to obtain menstrual materials to absorb or collect her menstrual blood*?
[Note: Write the list of responses on the flipchart paper in black ink]

Identify challenges: "*Ok, we have discussed what Mim can do to obtain menstrual materials. Now I want you to think about Mim or perhaps other friends you know. Can you tell me what might make it difficult for Mim or other girls to do each of these things?"* **[Note: Write the list of responses on the flipchart paper in red ink]**

Rate challenges: [Pass out stickers] "*Everyone has stickers in 2 colors: green and red. I want you to come to the flipchart and put your green sticker next to the condition you think would make it the MOST difficult to obtain menstrual materials. Place your red sticker next to the SECOND most difficult."*

Identify facilitators: "*Now I want you to think about Mim and other friends you know. Let us list some situations that would make it* ***easier*** *for Mim to obtain menstrual materials.* **[Note: Write the list of responses on the flipchart paper in green ink]**

Rate facilitators: "*Now come and place a green sticker next to the condition that would make doing these things easiest, and your red sticker beside the second easiest."*

[Repeat activity for other categories or potential sub-domains of the construct]
