# Supplementary material for: Development and validation of the Self-Efficacy in Addressing Menstrual Needs Scale (SAMNS-26) in Bangladeshi schools: A measure of girls’ menstrual care confidence
Source: PLoS One. 2022 Oct 6;17(10):e0275736. doi: 10.1371/journal.pone.0275736 (PMC9536616; doi:10.1371/journal.pone.0275736)
Supplement: S1 Table — (PDF) [file pone.0275736.s003.pdf]

S1 Table. Socio-demographic information of focus group discussion and cognitive interview participants during the development of the Self-Efficacy in Addressing Menstrual Needs Scale in Bangladesh, 2017-2018

Participant characteristics	FGD participants n=51	Cognitive interview participants n=21
Class in school		
4	3 (6%)	0 (0%)
5	5 (10%)	1 (5%)
6	2 (4%)	3 (14%)
7	11 (22%)	3 (14%)
8	9 (18%)	8 (38%)
9	20 (39%)	5 (24%)
10	1 (2%)	1 (5%)
Age		
11	4 (8%)	1 (5%)
12	8 (16%)	4 (19%)
13	8 (16%)	5 (24%)
14	21 (41%)	3 (14%)
15	9 (18%)	6 (29%)
16	1 (2%)	2 (10%)
Months since menarche		
0-6	6 (12%)	0 (0%)
7-12	9 (18%)	7 (33%)
13-18	2 (4%)	3 (14%)
19-24	8 (16%)	4 (19%)
25-30	14 (27%)	2 (10%)
Over 30	12 (24%)	5 (24%)
Mother’s education		
None	15 (29%)	7 (33%)
1 to 5 years	9 (18%)	8 (38%)
6 to 8 years	10 (20%)	3 (14%)
9 to 10 years	9 (18%)	2 (10%)
Over 10 years	4 (8%)	0 (0%)
Unknown	4 (8%)	1 (5%)
Father’s education		
None	13 (25%)	4 (19%)
1 to 5 years	7 (14%)	6 (29%)
6 to 8 years	7 (14%)	6 (29%)
9 to 10 years	5 (10%)	4 (19%)
Over 10 years	6 (12%)	0 (0%)
Unknown	13 (25%)	1 (5%)
