# Supplementary material for: Development and validation of the Self-Efficacy in Addressing Menstrual Needs Scale (SAMNS-26) in Bangladeshi schools: A measure of girls’ menstrual care confidence
Source: PLoS One. 2022 Oct 6;17(10):e0275736. doi: 10.1371/journal.pone.0275736 (PMC9536616; doi:10.1371/journal.pone.0275736)
Supplement: S2 Table — (PDF) [file pone.0275736.s004.pdf]

S2 Table. Exemplar tasks across categories which girls enact to address their menstrual needs, As reported by schoolgirls in Bangladesh during focus group discussions for the development of the Self-Efficacy in Addressing Menstrual Needs Scale, 2017-2018

Exemplar tasks	Exemplar conditions making tasks more difficult to enact	Exemplar conditions making tasks easier to enact
Obtaining menstrual materials		
• Buy pads from a shop/pharmacy<br>• Gather clean cotton material from around the house to use as an absorbent<br>• Ask for money to purchase pads<br>• Ask mother, teacher, friends, school janitor, etc. for a menstrual material	• Shopkeeper is male<br>• Shopkeeper is older<br>• Males are nearby<br>• Mother is not at home	• Shopkeeper is female<br>• Shopkeeper is a relative<br>• School provides pads<br>• Mother or older sister provides money or pads
Using, changing, disposing, and cleaning menstrual materials		
• Dispose of used menstrual materials appropriately<br>• Change the menstrual material every 4-6 hours<br>• Peel off sticker from wings of pad and dispose of the wrapping<br>• Feel (or check) when menstrual material is fully soaked and change it accordingly<br>• Wash menstrual cloths with water, soap, and disinfectant and dry thoroughly	• Mother and/or other female relatives are absent from home<br>• Sanitation facilities lacking at school<br>• Brothers and father are present in home<br>• Family shares toilet facilities with other households	• Friendly relationship with mother<br>• Female teachers provide pads<br>• Waste disposal system is available<br>• A fixed place beside the home gets adequate sunlight<br>• Private room is available (away from males)
Keeping our bodies clean		
• Take a bath daily<br>• Use soap when washing<br>• Wash hands when changing menstrual materials<br>• Change and wash outer garments if they become bloodstained	• No water supply<br>• Lack of warm water<br>• Lack of soap<br>• Only one bathroom available at home<br>• Lack of washing facilities at school<br>• Cold temperatures during winter season	• Washing facilities available at school<br>• Warm water is available<br>• Have access to more than one washroom at home<br>• Soap and water always available
Asking others for help, advice, or support		
• Ask others how to use menstrual materials and where to dispose of them or wash/dry them<br>• Ask for advice on which type of menstrual material is best to use<br>• Ask for advice on how to reduce menstrual pain<br>• Ask someone to check whether you have bloodstained your outer garments<br>• Ask a friend to accompany you to the school office to obtain a pad<br>• Ask someone for a menstrual material if you don't have one of your own	• Mother or older sister is not at home<br>• Mother herself does not know how to reduce menstrual pain<br>• Having just reached menarche<br>• Friends refuse to assist<br>• Male teacher does not allow student to leave classroom<br>• Males are present	• Mother and older sister are available at home<br>• Class teacher is female<br>• Classmates are supportive<br>• School janitor is female and readily available<br>• Pads kept in school common room instead of the teachers' office
Reducing menstrual pain or discomfort		
• Take advice from doctor<br>• Lay down on bed<br>• Drink oral rehydration solution<br>• Drink plenty of water<br>• Drink warm water<br>• Apply hot or cold fomentation<br>• Drink holy water<br>• Eat nutritious foods<br>• Apply *pan* leaf smeared with mustard oil to abdomen, then burn the leaf	• No hot water available<br>• Medicine not available at school<br>• Having to ask father for medicine<br>• Older brother and father are at home<br>• Oral rehydration solution not available at school<br>• Lack of privacy at home<br>• Mother not available	• Mother provides medicine<br>• School has medicine available<br>• School has a place to rest<br>• Privacy available (away from males)<br>• Allowed to rest head on desk in classroom<br>• Family member is a doctor<br>• Having someone available with whom one feels comfortable discussing such issues

**Exemplar tasks**	**Exemplar conditions making tasks more difficult to enact**	**Exemplar conditions making tasks easier to enact**
Managing stress and anxiety		
• Discuss with friends<br>• Discuss menstrual problems with teacher<br>• Use a pad properly<br>• Keep an extra pad with you<br>• Ask a friend to accompany you to the washroom or to collect a menstrual material	• Friend laughs/teases<br>• Mother, teacher, friends are too busy to listen or give time<br>• Not having someone to be open with<br>• Mother is not close by<br>• Crowds at toilets	• Boys are provided education about menstruation<br>• Accessing school common room/toilet allowed any time<br>• Having someone available with whom one feels comfortable discussing such issues<br>• Pads made available in school common room
