# Supplementary material for: Development and validation of the Self-Efficacy in Addressing Menstrual Needs Scale (SAMNS-26) in Bangladeshi schools: A measure of girls’ menstrual care confidence
Source: PLoS One. 2022 Oct 6;17(10):e0275736. doi: 10.1371/journal.pone.0275736 (PMC9536616; doi:10.1371/journal.pone.0275736)
Supplement: S3 Table — (PDF) [file pone.0275736.s005.pdf]

S3 Table: Characteristics of post-menarcheal schoolgirls who participated in a survey to test items for the development of the Self-Efficacy in Addressing Menstrual Needs Scale in Bangladesh, 2018

n=381 girls responding to the self-efficacy sub-study survey (n=200 urban, n=181 rural)

	**Urban**	**Rural**	**Combined**
	Mean (SD) or n (%)	Mean (SD) or n (%)	Mean (SD) or n (%)
Age	14.0 (1.5)	14.2 (1.4)	14.1 (1.5)
Age at menarche	11.5 (1.1)	12.1 (1.1)	11.8 (1.2)
Time since menarche (in years)	2.5 (1.4)	2.1 (1.2)	2.3 (1.3)
Mother's education			
None	14 (7.0%)	22 (12.2%)	36 (9.5%)
1-5 years	34 (17.0%)	54 (29.8%)	88 (23.1%)
6-8 years	27 (13.5%)	29 (16.0%)	56 (14.7%)
9-10 years	53 (26.5%)	47 (26.0%)	100 (26.3%)
Over 10 years	46 (23.0%)	8 (4.4%)	54 (14.2%)
Unknown	26 (13.0%)	21 (11.6%)	47 (12.3%)
Father's education			
None	12 (6.0%)	33 (18.2%)	45 (11.8%)
1-5 years	16 (8.0%)	18 (9.9%)	34 (8.9%)
6-8 years	17 (8.5%)	25 (13.8%)	42 (11.0%)
9-10 years	44 (22.0%)	29 (16.0%)	73 (19.2%)
Over 10 years	60 (30.0%)	30 (16.6%)	90 (23.6%)
Unknown	51 (25.5%)	46 (25.4%)	97 (25.5%)
Menstrual materials used while at home during last menstrual period (*multiple options possible*)			
Piece of cloth (new or repurposed)	40 (20.0%)	53 (29.3%)	93 (24.4%)
Disposable sanitary pad	129 (64.5%)	106 (58.6%)	235 (61.7%)
Reusable sanitary pad	62 (31.0%)	67 (37.0%)	129 (33.9%
Tissue	18 (9.0%)	7 (3.9%)	25 (6.6%)
Other	4 (2.0%)	0 (0.0%)	4 (1.1%)
Menstrual materials used while away from home during last menstrual period (*multiple options possible*)			
Piece of cloth (new or repurposed)	23 (11.5%)	35 (19.3%)	58 (15.2%)
Disposable sanitary pad	145 (72.5%)	123 (68.0%)	268 (70.3%)
Reusable sanitary pad	51 (25.5%)	48 (26.5%	99 (26.0%)
Tissue	14 (7.0%)	5 (2.8%)	19 (5.0%)
Other	2 (1.0%)	1 (0.6%)	3 (0.8%)
Genital washing during last menstrual period			
Didn't wash daily	2 (1.0%)	8 (4.4%)	10 (2.6%)
Once per day	3 (1.5%)	2 (1.1%)	5 (1.31%)
More than once per day	195 (97.5%)	171 (94.5%)	366 (96.1%)
Frequency of changing menstrual materials during last menstrual period			
Number of times change per day	4.0 (1.7)	3.7 (1.0)	3.9 (1.4)
Number of times changed per night	0.3 (0.8)	0.3 (0.5)	0.3 (0.6)
Maximum hours gone between changing	7.7 (3.5)	8.8 (3.1)	8.2 (3.4)

	Urban	Rural	Combined
	Mean (SD) or n (%)	Mean (SD) or n (%)	Mean (SD) or n (%)
Method of washing menstrual materials during last menstrual period (*multiple options possible*)*			
Water only	17 (16.8%)	10 (8.9%)	27 (12.6%)
Water and soap	89 (88.1%)	102 (90.3%)	191 (89.3%)
Water and disinfectant	9 (8.9%)	3 (2.7%)	12 (5.6%)
Water, soap, and disinfectant	7 (6.9%)	9 (8.0%)	16 (7.5%)
Location of drying menstrual materials during last menstrual period (*multiple options possible*)*			
Hidden inside room	11 (10.9%)	8 (7.08%)	19 (8.88%)
Open place inside room	21 (20.8%)	20 (17.70%)	41 (19.16%)
Outside in sunlight	54 (53.5%)	74 (65.49%)	128 (59.81%)
Outside but hidden	9 (8.9%)	9 (7.96%)	18 (8.41%)
Other (e.g. kitchen, toilet, bathroom)	3 (3.0%)	3 (2.65%)	6 (2.80%)
Directly underneath other clothes being dried	7 (6.9%)	5 (4.42%)	12 (5.61%)
Disposal method for those using a disposable menstrual material during last menstrual period (*multiple options possible*)+			
Open space	3 (1.88%)	1 (0.8%)	4 (1.4%)
In the bush	0 (0.0%)	18 (13.4%)	18 (6.1%)
In toilet pan	11 (6.88%)	31 (23.1%)	42 (14.3%)
In canal	2 (1.25%)	22 (16.4%)	24 (8.2%)
In waste bin	151 (96.88%)	32 (23.9%)	183 (62.2%)
Buried	0 (0.00%)	61 (45.5%)	61 (20.8%)
Other	0 (0.00%)	1 (0.8%)	1 (0.3%)
Location of changing menstrual materials during last menstrual period (*multiple options possible*)			
In toilet	35 (17.5%)	64 (35.4%)	99 (26.0%)
In bathroom	178 (89.0%)	66 (36.5%)	244 (64.0%)
In main (living) room	12 (6.0%)	71 (39.2%)	83 (21.8%)
Separate changing room	0 (0.0%)	12 (6.6%)	12 (3.2%)
Other	0 (0.0%)	2 (1.1%)	2 (0.5%)
Worried about being observed while changing menstrual materials during last menstrual period	23 (11.5%)	19 (10.5%)	42 (11.0%)

	Urban	Rural	Combined
	Mean (SD) or n (%)	Mean (SD) or n (%)	Mean (SD) or n (%)
Storage location for reusable menstrual materials between menstrual periods*			
Normally like other clothes	56 (55.5%)	45 (39.8%)	101 (47.2%)
Hidden place	39 (38.6%)	63 (55.8%)	102 (47.7%)
Under bed	3 (3.0%)	0 (0.0%)	3 (1.4%)
Other	3 (3.0%)	5 (4.4%)	8 (3.7%)
Wrapped reusable menstrual materials in polythene for next use*			
No	29 (28.7%)	24 (21.2%)	53 (24.8%)
Yes	72 (71.3%)	87 (77.0%)	159 (74.3%)
Don’t know	0 (0.0%)	2 (1.8%)	2 (0.9%)
Experienced pain during most recent menstrual period	135 (67.5%)	118 (65.2%)	253 (66.4%)
Severity of pain			
Mild	30 (22.2%)	30 (25.4%)	60 (23.7%)
Moderate	49 (36.3%)	40 (33.9%)	89 (35.2%)
Severe	56 (41.5%)	48 (40.7%)	104 (41.1%)
How long pain lasted			
1 day	73 (54.1%)	67 (56.8%)	140 (55.3%)
2 days	36 (26.7%)	31 (26.3%)	67 (26.5%)
3 days	23 (17.0%)	19 (16.1%)	42 (16.6%)
More than 3 days	3 (2.2%)	1 (0.9%)	4 (1.6%)

*Out of n=214 girls who kept menstrual materials to be reused (n=101 urban, n=113 rural)

+Out of n=294 girls who disposed of any menstrual material (n=160 urban, n=134 rural)
