# Supplementary material for: Development and validation of the Self-Efficacy in Addressing Menstrual Needs Scale (SAMNS-26) in Bangladeshi schools: A measure of girls’ menstrual care confidence
Source: PLoS One. 2022 Oct 6;17(10):e0275736. doi: 10.1371/journal.pone.0275736 (PMC9536616; doi:10.1371/journal.pone.0275736)
Supplement: S4 Table — (PDF) [file pone.0275736.s006.pdf]

S4 Table. Item response mean, standard deviation, skew, and kurtosis for 34 formally items tested with schoolgirls (n=381) for the development of the Self-Efficacy in Addressing Menstrual Needs Scale in Bangladesh, 2018

Abbreviated item	Frequency distribution chart	Mean	Standard deviation	Skewness	Kurtosis
SE1: … whenever it's time to change your menstrual material, you're able to find a place to do so where no one can see you	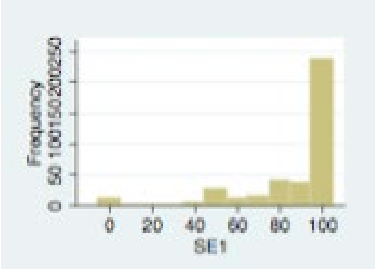	87.32283	22.58722	-2.244944	7.992063
SE2: Imagine you are at a relative's home and it becomes necessary to change the menstrual material you're wearing… you can change it there	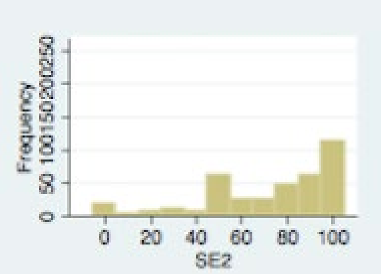	73.04462	27.68565	-.9930208	3.25343
SE3: … you can change your menstrual material at school if it becomes necessary	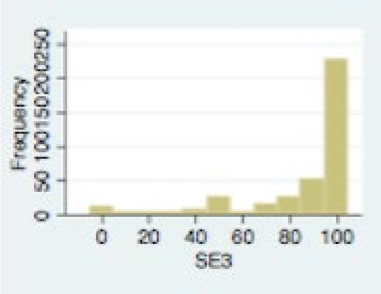	85.82677	24.63761	-2.008164	6.349751
SE4: … you can change your menstrual material if it becomes necessary at school even when the school toilet has no lock	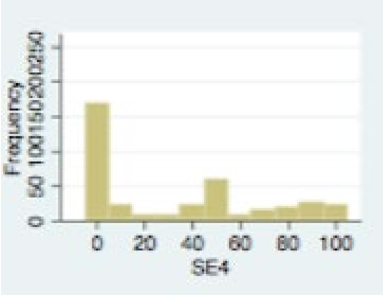	32.12598	35.13051	.5945266	1.904749
SE5: … you can change your menstrual material if it becomes necessary while you're at a female friend's house	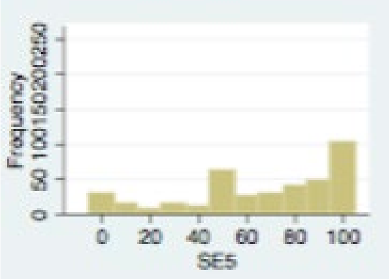	66.95538	31.44202	-.7236148	2.456402

SE6: When you put on a menstrual material, you are able to predict how long it will last before you will need to change it again	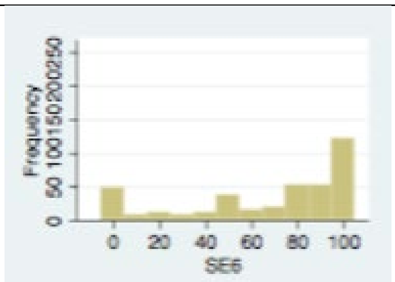	68.47368	34.5386	-.8935283	2.440506
SE7: … you can properly use a menstrual material so that menstrual blood does not stain your clothing while participating in school sports	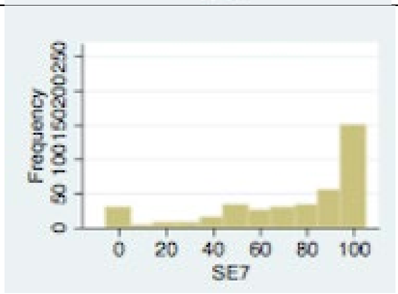	74.93438	30.46669	-1.19502	3.406151
SE8: … you can participate in your normal daily activities during your period without worry that your menstrual material will become displaced	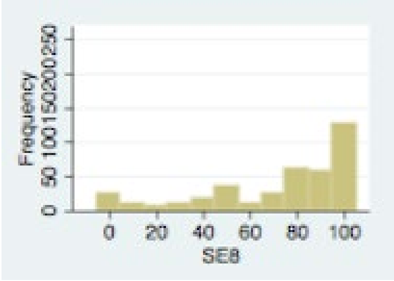	73.51706	30.55139	-1.149972	3.20855
SE9: …you can walk quickly during your period without your menstrual material becoming displaced	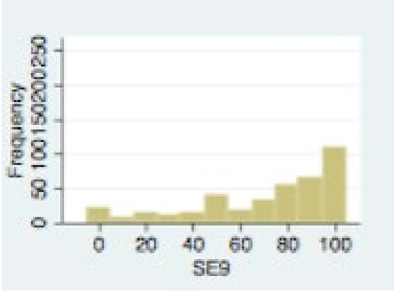	72.20472	29.71567	-1.045744	3.074665
SE10: … during your period, you can avoid bloodstaining your clothing while sitting for a 2-3 hour exam	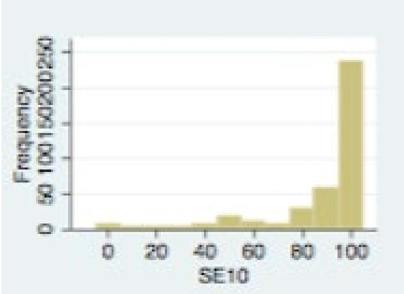	88.53018	21.60512	-2.393555	8.562732
SE11: If the menstrual material that you use most often is not available…you can use another type of menstrual material instead	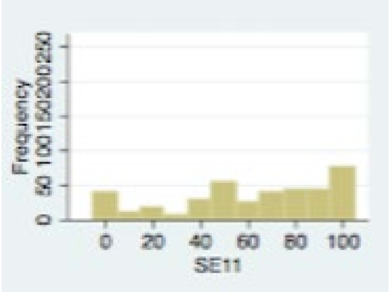	61.88976	32.03778	-.558177	2.218902

SE12: … you can lie down during your period without bloodstaining the bed sheet during the night	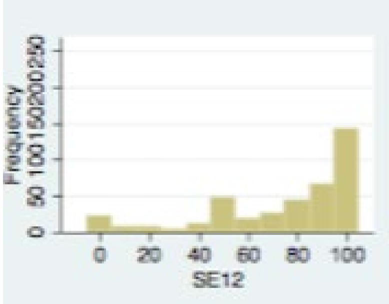	75.74803	29.15981	-1.249282	3.637324
SE13: … you are able to **try** to reduce abdominal pain during your period if it becomes necessary	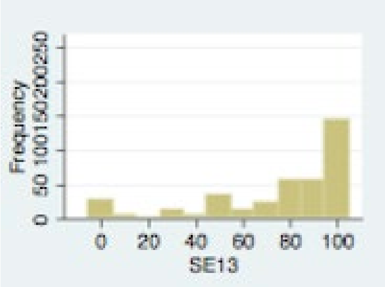	75.48556	30.15902	-1.287942	3.610238
SE14: … you can reduce abdominal pain during your period	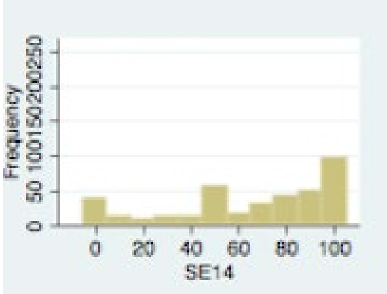	65.06562	33.33733	-.6988663	2.273064
SE15: … you can do hot fomentation if it becomes necessary to reduce abdominal pain	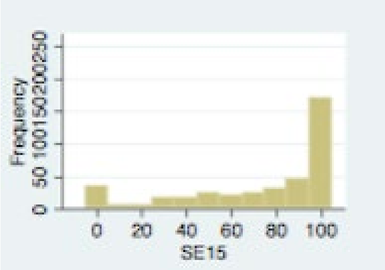	74.40945	32.64611	-1.140259	3.02964
SE16: … you can ask an adult for advice on how to reduce abdominal pain during your period	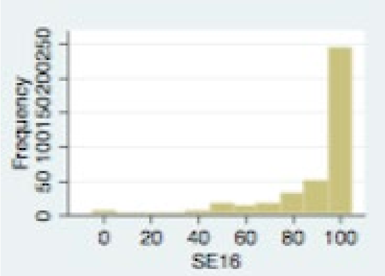	89.18635	19.95045	-2.366554	8.755558
SE17: … you can dispose of a used menstrual material if a male person is nearby	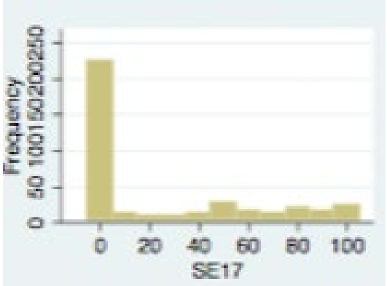	24.7769	34.569	1.007618	2.473561

SE18: Imagine you have gotten blood on your outer garments at school…you can remove the stain at school with having to return home	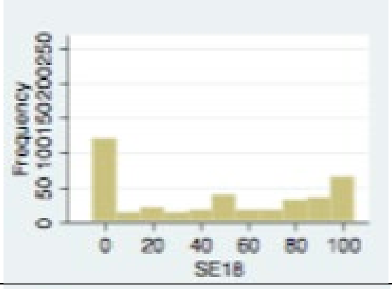	46.48294	39.21221	.0521607	1.418493
SE19: … you are at school and your period starts but you have not brought your own menstrual material…you are able to obtain a menstrual material somehow in that moment to meet your need while still at school	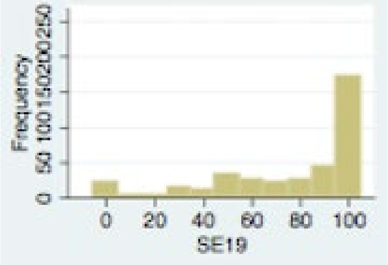	76.48294	30.00382	-1.186485	3.34592
SE20: …if necessary, you're able to ask a female friend for a menstrual material	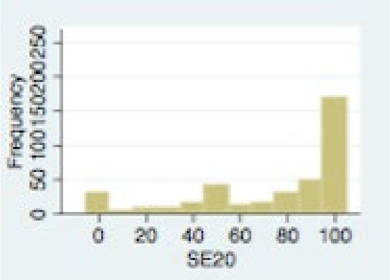	74.88189	31.77717	-1.121917	3.056526
SE21: … you can take help from a female teacher if you face a menstrual-related problem at school	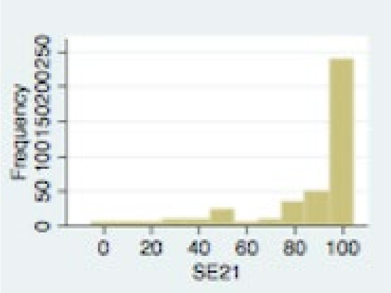	87.95276	21.84895	-2.135431	6.999084
SE22: … you can ask *aya* for help regarding your menstruation if a male teacher is nearby	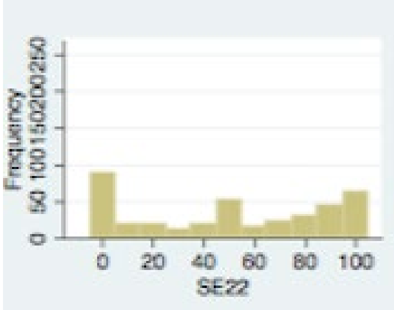	50.8399	37.77813	-.1227059	1.507402
SE23: Imagine pads are available at school…you can go ask for a pad by yourself when you need it, without the help of friends	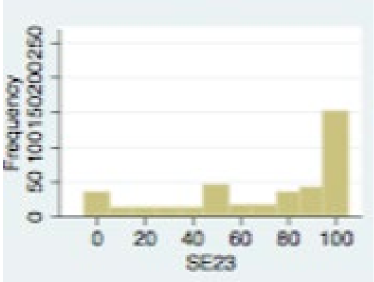	70.68241	33.8737	-.8983385	2.471409

SE24: Imagine you have the money to purchase a pad…you can ask a male seller at a pharmacy for a pad	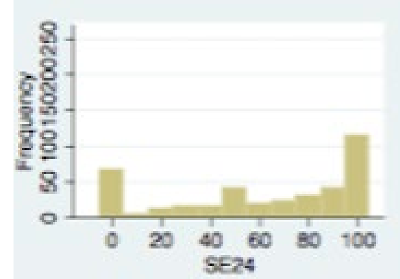	61.65354	37.1575	-.517292	1.816475
SE25: Imagine you have the money to purchase a pad…you can ask a pharmacy seller for a pad when there are male persons around	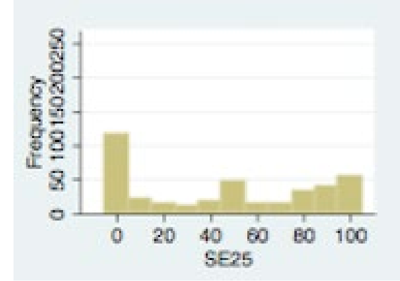	44.90814	38.6183	.1103216	1.449717
SE26: … you can roughly predict when your period is about to start	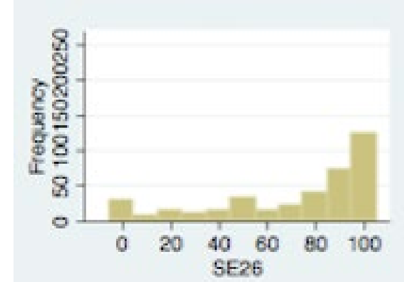	72.44094	31.87269	-1.07843	2.933886
SE27: … you are able to prevent bloodstaining your clothing even while traveling a long distance during your period	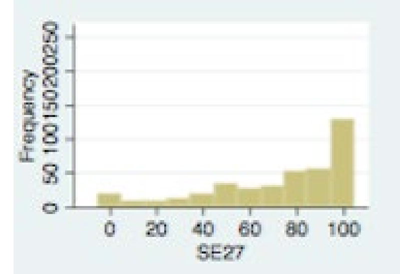	74.61942	28.45448	-1.136135	3.422933
SE28: … if Sir/Madam asks a question in class, you can stand up to answer during your period without worry that you have bloodstained your clothing	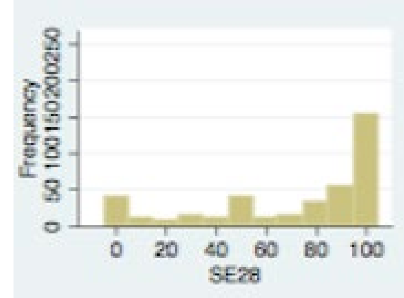	71.52231	34.40919	-.9767888	2.559875
SE29: … when you need menstrual materials you can obtain them even if a trusted female is not available at home	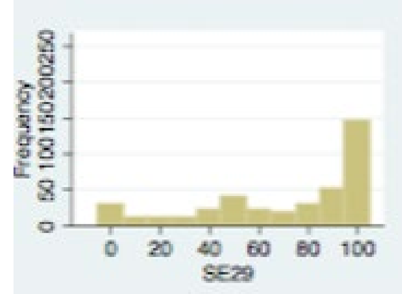	72.30971	32.14144	-.9639502	2.711061

SE30: … you can count/keep track of your period days	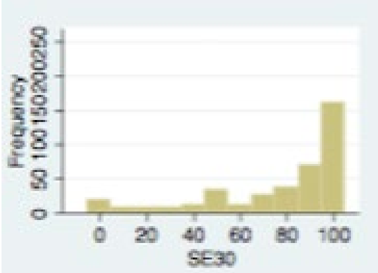	78.95013	28.09664	-1.459215	4.196615
SE31: … you can keep a menstrual material in your school bag around the time your period might start, so you can use them if you suddenly start bleeding	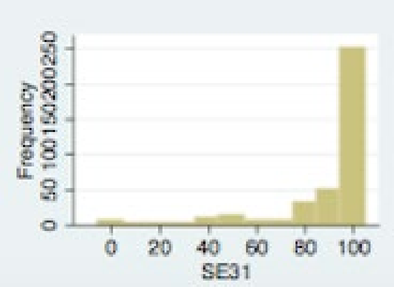	89.18635	21.53625	-2.515764	9.076215
SE32: … you can usually reduce your abdominal pain by a **small amount**	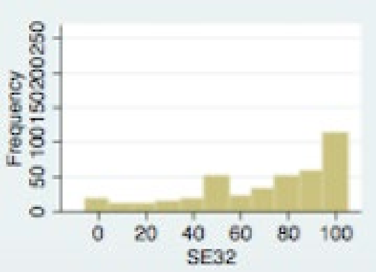	71.41732	29.21043	-.9235748	2.901624
SE33: … you can usually reduce **most** of your abdominal pain	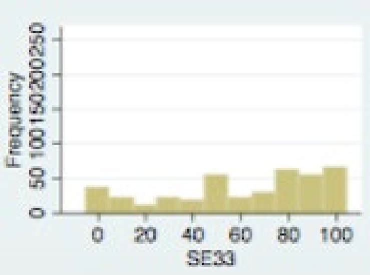	61.83727	32.51372	-.5785418	2.113024
SE34: … you can usually reduce your abdominal pain **completely**	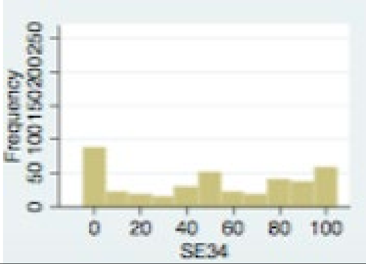	49.50131	36.73537	-.0705756	1.565957
