# Supplementary material for: Development and validation of the Self-Efficacy in Addressing Menstrual Needs Scale (SAMNS-26) in Bangladeshi schools: A measure of girls’ menstrual care confidence
Source: PLoS One. 2022 Oct 6;17(10):e0275736. doi: 10.1371/journal.pone.0275736 (PMC9536616; doi:10.1371/journal.pone.0275736)
Supplement: S5 Table — (PDF) [file pone.0275736.s007.pdf]

S5 Table. Items dropped during psychometric analyses of responses from 381 post-menarcheal schoolgirls in Bangladesh during the testing of the Self-Efficacy in Addressing Menstrual Needs Scale, 2018

**Dropped item**	**Rationale for dropping**
SE1: … whenever it's time to change your menstrual material, you're able to find a place to do so where no one can see you	Very low variability in responses
SE4: … you can change your menstrual material if it becomes necessary at school even when the school toilet has no lock	Did not correlate at least 0.30 with any other items
SE6: When you put on a menstrual material, you are able to predict how long it will last before you will need to change it again	Did not correlate at least 0.30 with any other items
SE10: … during your period, you can avoid bloodstaining your clothing while sitting for a 2-3 hour exam	Very low variability in responses
SE15: … you can do hot fomentation if it becomes necessary to reduce abdominal pain	Contributed poorly to internal reliability of menstrual pain management sub-scale
SE16: … you can ask an adult for advice on how to reduce abdominal pain during your period	Very low variability in responses
SE18: Imagine you have gotten blood on your outer garments at school…you can remove the stain at school with having to return home	Did not load at least 0.30 on any factor during EFA
SE31: … you can keep a menstrual material in your school bag around the time your period might start, so you can use them if you suddenly start bleeding	Very low variability in responses
