# Supplementary material for: Development and validation of the Self-Efficacy in Addressing Menstrual Needs Scale (SAMNS-26) in Bangladeshi schools: A measure of girls’ menstrual care confidence
Source: PLoS One. 2022 Oct 6;17(10):e0275736. doi: 10.1371/journal.pone.0275736 (PMC9536616; doi:10.1371/journal.pone.0275736)
Supplement: S1 File — (PDF) [file pone.0275736.s008.pdf]

MHM Self-Efficacy Scale Questionnaire (Version 05-April-2018)

Participant ID:

Date: ____________________

Start time: End time:

নিম্নোক্ত প্রশ্নগুলোতে মাসিকের সাথে সম্পর্কিত বিভিন্ন কার্যক্রমের তালিকা দেয়া হয়েছে। প্রতিটি প্রশ্নের জন্য আমি তোমার আত্মবিশ্বাসের মান নির্ধারন করতে চাই, এই কাজগুলো তুমি কতটা আত্মবিশ্বাসের সাথে এখন করতে পারছো। আত্মবিশ্বাসের মান নির্ধারণ করার জন্য নীচে দেওয়া স্কেল ব্যবহার করে ০ থেকে ১০০ পর্যন্ত একটি সংখ্যা নির্বাচন করে বৃত্ত আঁকো।

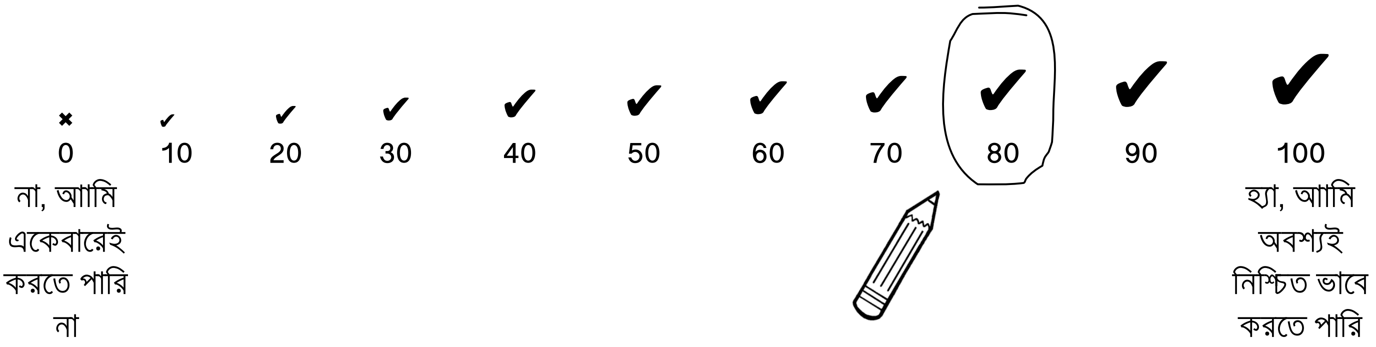

*নোট:

১। কেউ কোন প্রশ্ন বুঝতে না পারলে হাত তুলবে।

২। তোমাদের স্কুলের কেউ তোমার উত্তর দেখবে না সুতরাং নির্ভয়ে উত্তর দাও। এখানে কোন ভুল বা শুদ্ধ উত্তর নাই, তোমার যেটা মনে হয় সেটাতেই বৃত্ত আঁক।

১। তুমি কতটা আত্মবিশ্বাসী যে, মাসিকের উপকরণ (যেমনঃ প্যাড, কাপড়, টিসু, তুলা, ইত্যাদি) পরিবর্তন করার জন্য সবসময় এমন একটি জায়গা খুঁজে বের করতে পারো, যেখানে কেউ তোমাকে দেখবে না?

✖	✔	✔	✔	✔	✔	✔	✔	✔	✔	✔
0	10	20	30	40	50	60	70	80	90	100
না, আমি একেবারেই করতে পারি না										হ্যা, আমি অবশ্যই নিশ্চিত ভাবে করতে পারি

২। মনে কর তোমার কোন আত্মীয়ের বাসায় তোমার পরনের মাসিকের উপকরন (যেমনঃ কাপড়, প্যাড, তুলা, টিসু, ইত্যাদি)পরিবর্তন করার প্রয়োজন হলো। তুমি কতটা আত্মবিশ্বাসী যে, তুমি সেখানে এই কাজটি করতে পারো?

✖	✔	✔	✔	✔	✔	✔	✔	✔	✔	✔
0	10	20	30	40	50	60	70	80	90	100
না, আমি একেবারেই করতে পারি না										হ্যা, আমি অবশ্যই নিশ্চিত ভাবে করতে পারি

৩। তুমি কতটা আত্মবিশ্বাসী যে, স্কুলে মাসিকের উপকরণ (যেমনঃ প্যাড, কাপড়, টিসু, তুলা, ইত্যাদি) পরিবর্তন করার প্রয়োজন হলে তুমি তা স্কুলেই করতে পারো (স্কুল ছেড়ে না গিয়ে)?

✖	✔	✔	✔	✔	✔	✔	✔	✔	✔	✔
0	10	20	30	40	50	60	70	80	90	100
না, আমি একেবারেই করতে পারি না										হ্যা, আমি অবশ্যই নিশ্চিত ভাবে করতে পারি

৪। তুমি কতটা আত্মবিশ্বাসী যে, স্কুলের টয়লেটে কোন ছিটকিনি না থাকলেও মাসিকের উপকরণ (যেমনঃপ্যাড, কাপড়, টিসু, তুলা, ইত্যাদি) পরিবর্তন করার প্রয়োজন হলে তুমি তা করতে পারো?

✖	✔	✔	✔	✔	✔	✔	✔	✔	✔	✔
0	10	20	30	40	50	60	70	80	90	100
না, আমি একেবারেই করতে পারি না										হ্যা, আমি অবশ্যই নিশ্চিত ভাবে করতে পারি

৫। তুমি কতটা আত্মবিশ্বাসী যে, তোমার কোন বান্ধবীর বাসায় (তোমার নিজের বাসায় না গিয়ে) মাসিকের উপকরণ (যেমনঃ প্যাড, কাপড়, টিসু, তুলা, ইত্যাদি) পরিবর্তন করার প্রয়োজন হলে তুমি তা করতে পারো?

✖	✔	✔	✔	✔	✔	✔	✔	✔	✔	✔
0	10	20	30	40	50	60	70	80	90	100
না, আমি একেবারেই করতে পারি না										হ্যা, আমি অবশ্যই নিশ্চিত ভাবে করতে পারি

৬। তুমি কতটা আত্মবিশ্বাসী যে, তুমি যখন কোন মাসিকের উপকরন পরো (যেমনঃ প্যাড, কাপড়, টিসু, তুলা, ইত্যাদি) তখন আগে থেকেই বুঝতে পারো যে এটা কত সময় স্থায়ী হবে?

✖	✔	✔	✔	✔	✔	✔	✔	✔	✔	✔
0	10	20	30	40	50	60	70	80	90	100
না, আমি একেবারেই করতে পারি না										হ্যা, আমি অবশ্যই নিশ্চিত ভাবে করতে পারি

৭। তুমি কতটা আত্মবিশ্বাসী যে, স্কুলের খেলায় অংশগ্রহণ করার সময় সঠিকভাবে মাসিকের উপকরণ (যেমনঃ প্যাড, কাপড়, টিসু, তুলা, ইত্যাদি) ব্যবহার করতে পারো যাতে করে জামা কাপড়ে মাসিকের দাগ না লাগে?

✖	✔	✔	✔	✔	✔	✔	✔	✔	✔	✔
0	10	20	30	40	50	60	70	80	90	100
না, আমি একেবারেই করতে পারি না										হ্যা, আমি অবশ্যই নিশ্চিত ভাবে করতে পারি

৮। তুমি কতটা আত্মবিশ্বাসী যে, মাসিক চলাকালীন সময়ে তোমার উপকরণ (যেমনঃ প্যাড, কাপড়, টিস্যু, তুলা, ইত্যাদি) সরে যেতে পারে এই দু:শ্চিন্তা না করে স্বাভাবিক দৈনন্দিন কাজকর্ম চালিয়ে নিতে পারো?

✖	✔	✔	✔	✔	✔	✔	✔	✔	✔	✔
0	10	20	30	40	50	60	70	80	90	100
না, আমি একেবারেই করতে পারি না										হ্যা, আমি অবশ্যই নিশ্চিত ভাবে করতে পারি

৯। তুমি কতটা আত্মবিশ্বাসী যে, মাসিক চলাকালীন সময় তোমার মাসিকের উপকরণ (যেমনঃ প্যাড, কাপড়, টিসু, তুলা ইত্যাদি) সরে না গিয়ে দ্রুত হাঁটতে পারো?

✖	✔	✔	✔	✔	✔	✔	✔	✔	✔	✔
0	10	20	30	40	50	60	70	80	90	100
না, আমি একেবারেই করতে পারি না										হ্যা, আমি অবশ্যই নিশ্চিত ভাবে করতে পারি

১০। তুমি কতটা আত্মবিশ্বাসী যে, মাসিক চলাকালীন সময়ে তুমি এমন ভাবে মাসিকের উপকরন (যেমনঃ প্যাড, কাপড়, টিসু, তুলা ইত্যাদি.) পরতে পারো যাতে করে পরীক্ষায় ২-৩ ঘন্টা বসে থাকলেও তোমার জামা কাপড়ে মাসিকের রক্তের দাগ লাগবেনা।

✖	✔	✔	✔	✔	✔	✔	✔	✔	✔	✔
0	10	20	30	40	50	60	70	80	90	100
না, আমি একেবারেই করতে পারি না										হ্যা, আমি অবশ্যই নিশ্চিত ভাবে করতে পারি

১১। তুমি বেশীভাগ সময় মাসিকের যে উপকরনটি ব্যাবহার কর তা না পাওয়া গেলে, তুমি কতটা আত্মবিশ্বাসী যে (যেমনঃ প্যাড, কাপড়, টিসু, তুলা, ইত্যাদি) অন্য কোন উপকরণ ব্যবহার করতে পারো?

✖	✔	✔	✔	✔	✔	✔	✔	✔	✔	✔
0	10	20	30	40	50	60	70	80	90	100
না, আমি একেবারেই করতে পারি না										হ্যা, আমি অবশ্যই নিশ্চিত ভাবে করতে পারি

১২। তুমি কতটা আত্মবিশ্বাসী যে, মাসিক চলাকালীন সময় বিছানার চাদরে যেন দাগ না লাগে, রাত সেভাবে শুতে পারো?

✖	✔	✔	✔	✔	✔	✔	✔	✔	✔	✔
0	10	20	30	40	50	60	70	80	90	100
না, আামি একেবারেই করতে পারি না										হ্যা, আামি অবশ্যই নিশ্চিত ভাবে করতে পারি

১৩। তুমি কতটা আত্মবিশ্বাসী যে, মাসিকের সময় পেট ব্যাথা কমানোর প্রয়োজন হলে পেট ব্যাথা কমানোর চেষ্টা করতে পারো?

✖	✔	✔	✔	✔	✔	✔	✔	✔	✔	✔
0	10	20	30	40	50	60	70	80	90	100
না, আামি একেবারেই করতে পারি না										হ্যা, আামি অবশ্যই নিশ্চিত ভাবে করতে পারি

১৪। তুমি কতটা আত্মবিশ্বাসী যে, মাসিকের সময় পেট ব্যাথা তুমি কমাতে পারো?

✖	✔	✔	✔	✔	✔	✔	✔	✔	✔	✔
0	10	20	30	40	50	60	70	80	90	100
না, আামি একেবারেই করতে পারি না										হ্যা, আামি অবশ্যই নিশ্চিত ভাবে করতে পারি

১৫। তুমি কতটা আত্মবিশ্বাসী যে, পেট ব্যাথা কমানোর জন্য প্রয়োজনে গরম স্যাক নিতে পারো?

✖	✔	✔	✔	✔	✔	✔	✔	✔	✔	✔
0	10	20	30	40	50	60	70	80	90	100
না, আামি একেবারেই করতে পারি না										হ্যা, আামি অবশ্যই নিশ্চিত ভাবে করতে পারি

১৬। তুমি কতটা আত্মবিশ্বাসী যে, মাসিক চলাকালীন পেট ব্যাথা কমাতে বড়দের কাছ থেকে পরামর্শ চাইতে পারো?

✖	✔	✔	✔	✔	✔	✔	✔	✔	✔	✔
0	10	20	30	40	50	60	70	80	90	100
না, আমি একেবারেই করতে পারি না										হ্যা, আমি অবশ্যই নিশ্চিত ভাবে করতে পারি

১৭। তুমি কতটা আত্মবিশ্বাসী যে, একজন পুরুষ মানুষের উপস্থিতিতে তোমার ব্যবহৃত মাসিকের উপকরণ (যেমনঃ প্যাড, কাপড়, টিস্যু, তুলা, ইত্যাদি) ফেলতে পারো?

✖	✔	✔	✔	✔	✔	✔	✔	✔	✔	✔
0	10	20	30	40	50	60	70	80	90	100
না, আমি একেবারেই করতে পারি না										হ্যা, আমি অবশ্যই নিশ্চিত ভাবে করতে পারি

১৮। মনে কর স্কুলে তোমার জামায় রক্ত লেগেছে। তুমি কতটা আত্মবিশ্বাসী যে, বাসায় না গিয়ে স্কুলে থেকেই এই দাগ উঠাতে পারো?

✖	✔	✔	✔	✔	✔	✔	✔	✔	✔	✔
0	10	20	30	40	50	60	70	80	90	100
না, আমি একেবারেই করতে পারি না										হ্যা, আমি অবশ্যই নিশ্চিত ভাবে করতে পারি

১৯। ধরো, স্কুলে তোমার মাসিক শুরু হয়েছে এবং তুমি নিজের মাসিকের উপকরণ (যেমনঃ প্যাড, কাপড়, টিস্যু, তুলা, ইত্যাদি) নিয়ে আসনি। তুমি কতটা আত্মবিশ্বাসী যে, স্কুলে থাকা অবস্থায় ঐ সময় যে কোনভাবে একটি মাসিকের উপকরণ যোগাড় করে তোমার প্রয়োজন মেটাতে পারো?

✖	✔	✔	✔	✔	✔	✔	✔	✔	✔	✔
0	10	20	30	40	50	60	70	80	90	100
না, আমি একেবারেই করতে পারি না										হ্যা, আমি অবশ্যই নিশ্চিত ভাবে করতে পারি

২০। তুমি কতটা আত্মবিশ্বাসী যে, প্রয়োজনে মাসিকের উপকরণ (যেমনঃ প্যাড, কাপড়, টিসু, তুলা, ইত্যাদি)বান্ধবীর কাছে চাইতে পারো?

✖	✔	✔	✔	✔	✔	✔	✔	✔	✔	✔
0	10	20	30	40	50	60	70	80	90	100
না, আমি একেবারেই করতে পারি না										হ্যা, আমি অবশ্যই নিশ্চিত ভাবে করতে পারি

২১। তুমি কতটা আত্মবিশ্বাসী যে, স্কুলে মাসিক সংক্রান্ত কোন সমস্যায় পড়লে ম্যাডাম এর কাছে সাহায্য নিতে পারো?

✖	✔	✔	✔	✔	✔	✔	✔	✔	✔	✔
0	10	20	30	40	50	60	70	80	90	100
না, আমি একেবারেই করতে পারি না										হ্যা, আমি অবশ্যই নিশ্চিত ভাবে করতে পারি

২২। তুমি কতটা আত্মবিশ্বাসী যে, স্যার আশেপাশে থাকলেও আয়ার কাছে মাসিকের জন্য সাহায্য চাইতে পারো?

✖	✔	✔	✔	✔	✔	✔	✔	✔	✔	✔
0	10	20	30	40	50	60	70	80	90	100
না, আমি একেবারেই করতে পারি না										হ্যা, আমি অবশ্যই নিশ্চিত ভাবে করতে পারি

a.

২৩। ধরো, স্কুলে প্যাড আছে, তুমি কতটা আত্মবিশ্বাসী যে, তোমার কোন বান্ধবীর সাহায্য ছাড়াই তুমি একা গিয়ে নিজের জন্য প্যাড চাইতে পারো?

✖	✔	✔	✔	✔	✔	✔	✔	✔	✔	✔
0	10	20	30	40	50	60	70	80	90	100
না, আমি একেবারেই করতে পারি না										হ্যা, আমি অবশ্যই নিশ্চিত ভাবে করতে পারি

২৪। ধরো, তোমার কাছে প্যাড কেনার টাকা আছে। তুমি কতটা আত্মবিশ্বাসী যে, একজন ফার্মাসীর পুরুষ দোকানদারের কাছে প্যাড চাইতে পারো?

✖	✔	✔	✔	✔	✔	✔	✔	✔	✔	✔
0	10	20	30	40	50	60	70	80	90	100
না, আমি একেবারেই করতে পারি না										হ্যা, আমি অবশ্যই নিশ্চিত ভাবে করতে পারি

২৫। ধরো, তোমার কাছে প্যাড কেনার টাকা আছে। তুমি কতটা আত্মবিশ্বাসী যে, আশেপাশে পুরুষরা থাকলেও তুমি ফার্মাসীর দোকানদারের কাছে প্যাড চাইতে পারো?

✖	✔	✔	✔	✔	✔	✔	✔	✔	✔	✔
0	10	20	30	40	50	60	70	80	90	100
না, আমি একেবারেই করতে পারি না										হ্যা, আমি অবশ্যই নিশ্চিত ভাবে করতে পারি

২৬। তুমি কতটা আত্মবিশ্বাসী যে, আনুমানিক কবে তোমার মাসিক শুরু হবে তা আগে থেকেই আন্দাজ করতে পারো?

✖	✔	✔	✔	✔	✔	✔	✔	✔	✔	✔
0	10	20	30	40	50	60	70	80	90	100
না, আমি একেবারেই করতে পারি না										হ্যা, আমি অবশ্যই নিশ্চিত ভাবে করতে পারি

২৭। তুমি কতটা আত্মবিশ্বাসী যে, মাসিক চলাকালীন সময়ে যদি তোমাকে দূরে কোথাও ভ্রমনে যেতে হয়, তখন যাতায়াতের সময় তুমি তোমার জামায় দাগ লাগানো থেকে বিরত থাকতে পারো?

✖	✔	✔	✔	✔	✔	✔	✔	✔	✔	✔
0	10	20	30	40	50	60	70	80	90	100
না, আমি একেবারেই করতে পারি না										হ্যা, আমি অবশ্যই নিশ্চিত ভাবে করতে পারি

২৮। তুমি কতটা আত্মবিশ্বাসী যে, মাসিক চলাকালীন সময়ে ক্লাশে স্যার/ ম্যাডাম যদি প্রশ্ন করে তবে জামায় মাসিকের দাগ লাগার দুঃশ্চিন্তা না করে দাঁড়িয়ে প্রশ্নের উত্তর দিতে পারো?

✖	✔	✔	✔	✔	✔	✔	✔	✔	✔	✔
0	10	20	30	40	50	60	70	80	90	100
না, আমি একেবারেই করতে পারি না										হ্যা, আমি অবশ্যই নিশ্চিত ভাবে করতে পারি

২৯। তুমি কতটা আত্মবিশ্বাসী যে, যখন তোমার মাসিকের উপকরণ (যেমনঃ প্যাড, কাপড়, টিসু, তুলা, ইত্যাদি) দরকার হয় তখন যদি মহিলা আপনজন (যেমনঃ মা, বোন, ভাবী, ইত্যাদি) তোমার বাসায় না থাকে তাহলেও তুমি তা যোগাড় করতে পারো?

✖	✔	✔	✔	✔	✔	✔	✔	✔	✔	✔
0	10	20	30	40	50	60	70	80	90	100
না, আমি একেবারেই করতে পারি না										হ্যা, আমি অবশ্যই নিশ্চিত ভাবে করতে পারি

৩০। তুমি কতটা আত্মবিশ্বাসী যে, তুমি তোমার পরবর্তী মাসিকের দিন হিসাব করতে পারো?

✖	✔	✔	✔	✔	✔	✔	✔	✔	✔	✔
0	10	20	30	40	50	60	70	80	90	100
না, আমি একেবারেই করতে পারি না										হ্যা, আমি অবশ্যই নিশ্চিত ভাবে করতে পারি

৩১। তুমি কতটা আত্মবিশ্বাসী যে, মাসিক হতে পারে এমন দিনগুলির জন্য তুমি স্কুল ব্যাগে মাসিকের উপকরন (যেমনঃ কাপড়, প্যাড, টিস্যু, তুলা, ইত্যাদি) রাখতে পারো যেন হঠাৎ মাসিক হলে তুমি তা ব্যবহার করতে পারো?

✖	✔	✔	✔	✔	✔	✔	✔	✔	✔	✔
0	10	20	30	40	50	60	70	80	90	100
না, আামি একেবারেই করতে পারি না										হ্যা, আামি অবশ্যই নিশ্চিত ভাবে করতে পারি

৩২। তুমি কতটা আত্মবিশ্বাসী যে, তোমার মাসিকের পেট ব্যাথা সাধারনত তুমি কিছুটা কমাতে পারো?

✖	✔	✔	✔	✔	✔	✔	✔	✔	✔	✔
0	10	20	30	40	50	60	70	80	90	100
না, আামি একেবারেই করতে পারি না										হ্যা, আামি অবশ্যই নিশ্চিত ভাবে করতে পারি

৩৩। তুমি কতটা আত্মবিশ্বাসী যে, তোমার মাসিকের পেট ব্যাথা সাধারনত তুমি বেশীর ভাগ কমাতে পারো?

✖	✔	✔	✔	✔	✔	✔	✔	✔	✔	✔
0	10	20	30	40	50	60	70	80	90	100
না, আামি একেবারেই করতে পারি না										হ্যা, আামি অবশ্যই নিশ্চিত ভাবে করতে পারি

৩৪। তুমি কতটা আত্মবিশ্বাসী যে, তোমার মাসিকের পেট ব্যাথা সাধারনত তুমি সম্পূর্ণ কমাতে পারো?

✖	✔	✔	✔	✔	✔	✔	✔	✔	✔	✔
0	10	20	30	40	50	60	70	80	90	100
না, আামি একেবারেই করতে পারি না										হ্যা, আামি অবশ্যই নিশ্চিত ভাবে করতে পারি

This page intentionally left blank

তুমি এই সেকশনের উত্তর দেয়া শেষ করেই হাত তোল, আমি আসব।
