# Supplementary material for: Development and validation of the Self-Efficacy in Addressing Menstrual Needs Scale (SAMNS-26) in Bangladeshi schools: A measure of girls’ menstrual care confidence
Source: PLoS One. 2022 Oct 6;17(10):e0275736. doi: 10.1371/journal.pone.0275736 (PMC9536616; doi:10.1371/journal.pone.0275736)
Supplement: S2 File — (PDF) [file pone.0275736.s009.pdf]

S2 File. Description of survey measures included in the testing of the Self-Efficacy in Addressing Menstrual Needs Scale in Bangladesh, 2018

The self-efficacy sub-study survey had two sections: the first comprised 34 self-efficacy items for formal testing. The second section comprised three additional measures for use in the assessment of the SAMNS construct validity.

Self-efficacy in addressing menstrual needs

We tested 34 items for potential inclusion in the Self-Efficacy in Addressing Menstrual Needs Scale. Each item represented a task involved in addressing menstrual needs, and participants indicated how confident they felt they were able to accomplish the task. Likert-type response options (0-100 in intervals of 10) were provided with anchor wording at 0= "No, I absolutely cannot do it" and 100= "Yes, I am absolutely sure I can definitely do it." For analyses, responses and scale scores were treated as continuous.

Self-concept

The first validation measure was the Bengali version [1] of the Beck Self-Concept Inventory for Youth (BSCI-Y) [2], which was used to assess girls' cognitions of positive self-worth. The self-report measure consists of 20 items with a 4-point Likert-type response scale ranging from 0 (never) to 3 (always). The Bengali version had been previously validated for use with Bangladeshi adolescent girls, during which it was shown to have good internal reliability (Cronbach α= 0.77) in a sample of 87 girls and a 10-day test-retest reliability of 0.80 (N=29) [1]. Although often conflated, self-efficacy is a construct related to yet distinct from self-concept or self-worth [3]. Therefore, we expected not to see a strong correlation between the SAMNS and the BSCI-Y.

Anxiety

Girls completed the 20-item Bengali version [1] of the Beck Anxiety Inventory for Youth (BAI-Y) [2]. Like the BSCI-Y, the measure had a 4-point Likert-type response scale ranging from 0 (never) to 3 (always). The Bengali version of the BAI-Y had been previously validated for use with Bangladeshi adolescents, with internal reliability of 0.88 and test-retest reliability of 0.79 [1]. Informed by self-efficacy theory, we anticipated that SAMNS scores would be negatively correlated with measures of anxiety. However, since the BAI-Y is not a specific measure of menstrual-related state anxiety, we hypothesized that correlations would be low to moderate.

Social self-efficacy

We could not find a measure of social self-efficacy that had been previously validated for Bangladeshi adolescents; therefore we translated the Social Self-Efficacy Scale [4] from English into Bengali then back-translated and revised before pretesting in a sample of 13 schoolgirls to ensure easy comprehension. The 8-item measure assesses adolescents' beliefs of their capabilities to negotiate social situations and produce successful social interactions [4]. Each item asks participants to indicate with Likert-type response options ranging from 1 (not very well) to 5 (very well) how well they can do a particular task. Reponses to the eight items are summed to calculate a scale score. We included social self-efficacy as a validation measure because some of our SAMNS items involve interacting with and obtaining assistance from others to address menstrual needs. Although we hypothesized there would be a positive correlation between SAMNS scores and Social Self-Efficacy Scale scores, we wanted to ensure that our finalized measure was not so highly correlated with the construct of social self-efficacy that it would not provide any additional utility.

Additional validation items
Additional items from the main study's endline survey were used for validity testing. The items "During my last period, I felt anxious at school because of my menstruation" and "During my last period, I felt comfortable at school" served as indicators of anxious arousal more specifically related to menstrual experiences than the BAI-Y. The item "I prefer to stay at home during my period" was used to validate whether girls with lower self-efficacy scores tended to desire avoidance of contexts that make addressing menstrual needs challenging. Participants' responses to these items on a 6-point Likert-type response scale of 1= strongly agree to 6= strongly disagree were dichotomized as "agree" or "disagree" for the present analysis.

We hypothesized that girls' self-efficacy scores would positively correlate with time (months) since menarche, calculated from two items on the main study's survey: current age and age when menstruation first began.

References

1. Uddin M, Ul-Huque A, Shimul A. Adaptation of the Beck Youth Inventories of Emotional and Social Impairment for Use in Bangladesh. The Dhaka University Journal of Psychology. 2011;35:65-80.
2. Beck J, Beck A, Jolly J. Manual for the Beck Youth Inventories of Emotional and Social Impairment. San Antonio, TX: The Psychological Corporation; 2001.
3. Bandura A. Self-Efficacy: The Exercise of Control. New York: W.H. Freeman and Company; 1997.
4. Muris P. A Brief Questionnaire for Measuring Self-Efficacy in Youths. Journal of Psychopathology and Behavioral Assessment. 2001;23(3):145-9.
