# Supplementary material for: Development and validation of the Self-Efficacy in Addressing Menstrual Needs Scale (SAMNS-26) in Bangladeshi schools: A measure of girls’ menstrual care confidence
Source: PLoS One. 2022 Oct 6;17(10):e0275736. doi: 10.1371/journal.pone.0275736 (PMC9536616; doi:10.1371/journal.pone.0275736)
Supplement: S3 File — (PDF) [file pone.0275736.s010.pdf]

Self-Efficacy in Addressing Menstrual Needs Scale (SAMNS-26)
[English translation of original Bengali version]

Instructions and response options:

Each of the following questions describes an activity related to menstruation. For each question, rate your current level of confidence that you are able to do the task mentioned. To rate your level of confidence, use the scale provided by drawing a circle around a number from 0 to 100:

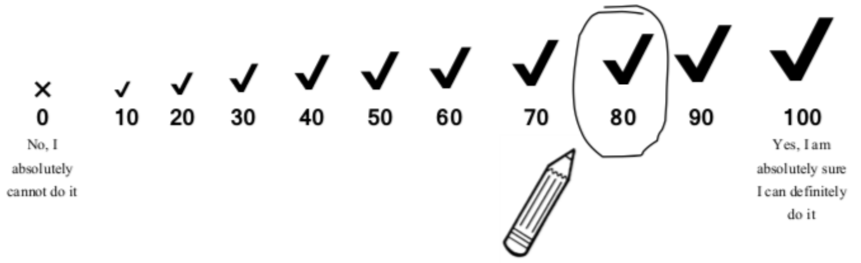

*Note:
1. If you do not understand a question, raise your hand
2. No one at your school will see your answers, so you can be comfortable to answer how you truly feel. There are no right or wrong answers; just circle the answer that best represents you.

Items:

1. Imagine you are at a relative's home and it becomes necessary to change the menstrual material you're wearing (such as: pad, cloth, tissue, cotton, etc.). How confident are you that you can change it there?

2. How confident are you that you can change your menstrual material (such as: pad, cloth, tissue, cotton, etc.) at school if it becomes necessary (*without leaving school*)?

3. How confident are you that you can change your menstrual material (such as: pad, cloth, tissue, cotton, etc.) if it becomes necessary while you're at a female friend's house (*without returning to your own home*)?

4. How confident are you that you can properly use a menstrual material (such as: pad, cloth, tissue, cotton, etc.) so that menstrual blood does not stain your clothing while participating in school sports?

5. How confident are you that you can participate in your normal daily activities during your period without worry that your menstrual material (such as: pad, cloth, tissue, cotton, etc.) will become displaced?

6. How confident are you that you can walk quickly during your period without your menstrual material (such as: pad, cloth, tissue, cotton, etc.) becoming displaced?

7. If the menstrual material that you use most often is not available, how confident are you that you can use another type of menstrual material (such as: pad, cloth, tissue, cotton, etc.) instead?

8. How confident are you that you can lie down during your period without bloodstaining the bed sheet during the night?

9. How confident are you that you are able to **try** to reduce abdominal pain during your period if it becomes necessary?

10. How confident are you that you can reduce abdominal pain during your period?

11. How confident are you that you can dispose of a used menstrual material (such as: pad, cloth, tissue, cotton, etc.) if a male person is nearby?

12. Take for instance that you are at school and your period starts but you have not brought your own menstrual material (such as: pad, cloth, tissue, cotton, etc.). How confident are you that you are able to obtain a menstrual material somehow in that moment to meet your need while still at school?

13. How confident are you that, if necessary, you're able to ask a female friend for a menstrual material (such as: pad, cloth, tissue, cotton, etc.)?

14. How confident are you that you can take help from a female teacher if you face a menstrual-related problem at school?

15. How confident are you that you can ask *aya** for help regarding your menstruation if a male teacher is nearby?

16. Imagine pads are available at school. How confident are you that you can go ask for a pad by yourself when you need it, without the help of friends?

17. Imagine you have the money to purchase a pad. How confident are you that you can ask a male seller at a pharmacy for a pad?

18. Imagine you have the money to purchase a pad. How confident are you that you can ask a pharmacy seller for a pad when there are male persons around?

19. How confident are you that you can roughly predict when your period is about to start?

20. How confident are you that you are able to prevent bloodstaining your clothing even while traveling a long distance during your period?

21. How confident are you that if Sir/Madam asks a question in class, you can stand up to answer during your period without worry that you have bloodstained your clothing?

22. How confident are you that when you need menstrual materials (such as: pad, cloth, tissue, cotton, etc.) you can obtain them even if a trusted female (such as: mother, sister, sister-in-law, etc.) is not available at home?

23. How confident are you that you can count/keep track of your period days?

24. How confident are you that you can usually reduce your abdominal pain by a **small amount**?

25. How confident are you that you can usually reduce **most** of your abdominal pain?

26. How confident are you that you can usually reduce your abdominal pain **completely**?

* *Aya* refers to women who work in the schools as janitors and caretakers. Schoolgirls in the scale development study schools typically had friendly relationships with these women, and if the school provided any menstrual pads for emergencies, it was typically the *aya* who could give one to a student.

Sub-Scales by item number:

Menstrual Hygiene Preparation and Maintenance Sub-Scale (MHPM): 1, 2, 3, 4, 5, 6, 7, 8, 12, 13, 14, 16, 19, 20, 21, 22, 23

Menstrual Pain Management Sub-Scale (MPM): 9, 10, 24, 25, 26

Executing Stigmatized Tasks Sub-Scale (EST): 11, 15, 17, 18

Scoring: No reverse scoring is required. Calculate SAMNS-26 sub-scale scores by calculating the sum of a respondent's responses across items and divide by total number of items. Scores can range from 0 to 100.

Protocol for administering SAMNS-26 with adolescent schoolgirls:

1. Data enumerator should provide verbal instructions to the respondent for completing the questionnaire: *"Each of the following questions describes an activity related to menstruation. For each question, I want you to rate your current level of confidence (in other words, how* ***sure*** *are you) that you are able to do the task mentioned. To rate your level of confidence that you can do the task, use the scale provided by drawing a circle around a number from 0 to 100 where "0" means "No, I absolutely cannot do it" and 100 means "Yes, I am absolutely sure I can definitely do it."* **[These instructions should also be printed at the top of the tool, and the Likert-type response options should be printed after each item with anchoring words at 0 and 100.]**

2. After providing verbal instructions to the respondent, the **data enumerator should do a quick test for comprehension.** To implement the test, the data enumerator places a pen on the table very near to a respondent and asks "*How confident are you that you can reach the pen (while remaining in your seat)*?" with the expectation that the response should be at or near 100. The data enumerator then moves the pen progressively farther away and repeatedly asks the respondent to select her level of confidence that she could reach the pen. If responses do not move in a reasonable manner along the response scale, then the data enumerator should identify the cause of error and provide further explanation of the instructions to clarify misunderstandings in how to respond to items.

3. Once the data enumerator is confident that the respondent is clear on the instructions and how to use the Likert-type response options correctly, s/he should instruct the respondent to complete the tool on her own, raising her hand should she have any questions or confusion while completing it.
