# Supplementary material for: Development and validation of the Self-Efficacy in Addressing Menstrual Needs Scale (SAMNS-26) in Bangladeshi schools: A measure of girls’ menstrual care confidence
Source: PLoS One. 2022 Oct 6;17(10):e0275736. doi: 10.1371/journal.pone.0275736 (PMC9536616; doi:10.1371/journal.pone.0275736)
Supplement: Attachment — Submitted filename: SAMNS-26.docx [file pone.0275736.s013.docx]

The paper describes the development of a new tool to assess psychosocial components of menstruation as experienced by adolescent schoolgirls. Evidence to date suggests that poor menstrual hygiene impacts negatively on girls education leading to school absence and drop-out, girls who remain in school whilst menstruating face issues such as inability to concentrate on their lessons, and period shaming. The negative impact on girls’ education contributes towards gender inequality. The tool described in this paper has been developed to assess the psychosocial components of schoolgirls menstruation and can therefore be used to develop interventions improving the experience of menses. It is likely that this could positively impact on girls education and go some way to help address gender imbalance in the future.

I would like to commend the authors on this well written paper. The development of the tool was very thorough. There is much detail provided in the paper and each step was very clearly described. This left me with few questions or sections where I needed further clarification.

One concern that I have is that whilst the authors are realistic and state that the tool was developed and validated only in the Bangladeshi context, and not intended to be a universal measure, this does weaken its relevance to PLOS One readers generally. I feel that the discussion is relatively weak compared to the rest of the paper, and one way of enhancing might be to provide more clarification on how the tool could be amended for wider use – without following all of the same procedures as described in this paper, which clearly involved much detailed fieldwork, in order to recreate a similar model. I appreciate that there are other issues involved in menstrual care that might be relevant to other contexts, but from my own experience in HIC and LIC settings the list of items in SAMNS-26 seems applicable generally, (bar the one item specific to aya, as highlighted by the authors). I appreciate that to some extent lines 604 – 625 do touch on this, as does line 656-657. Perhaps just to turn around the narrative a bit in order to be more positive and definitive about the use of the SAMNS-26 and how it could be taken forward or adapted, might open this out more to a wider audience. For example, perhaps line 655 could read along the lines of ‘Additional testing of the tool in the Bangladeshi and similar contexts is recommended…..’.

In addition to the above suggestion, I would like to see the discussion strengthened to do justice to the work that has been undertaken. In particular, the precis of the methods in the first couple of paragraphs is rather repetitive. This could be removed and replaced with a succinct statement of what this study has achieved with room then for more in-depth discussion of strengths and future use of the tool (and areas for development) incorporated instead.

I also would like to see the discussion incorporating more literature. Currently, it references just 3 studies but would benefit from greater alignment with a wider body of literature to confirm or refute study methods and / or findings and ways forward to assist management of menstrual hygiene. It might also be interesting to discuss the SAMNS-26 scores – I was particularly interested to note there were only small differences in score between those using disposable pads compared to other materials when away from home, (also that sub-scale scores, particularly Preparation and Task were not correlated with months since menarche. This is picked up on in the discussion but no references are given to assist the reader in understanding why you may have developed your hypothesis, nor to evidence your (reasonable) suggestion that time provides opportunity to diminish self-efficacy). These would strengthen your arguments made.

The introduction makes mention that individuals with low self-efficacy may (theoretically) avoid challenging situations such as attending school during menstruation. As repeated absence is linked with school drop-out would it be useful to mention perhaps as a limitation of the study, that it is school-based only and therefore may miss those girls who experience the greatest levels of menstrual related stress and anxiety?

I have little specific feedback, because as stated, I feel that the authors have written a paper that is of a good standard.

Abstract

Line 36 – add a few words to incorporate the importance of measuring psychosocial components or why it is needed

Line 62- the abstract conclusion suggests further research should explore whether secondary dysmenorrhoea impacts how girls interpret and respond to items. However, this is not mentioned as a conclusion in the main paper, although it is mentioned in the discussion. I suggest that it is removed from here, or if the authors think it is a key take home message, then it should be in the actual study conclusion.

Methods

Lines 138-153. I cannot easily reconcile the description provided (lines 138-145), nor the description of phase 1 (lines 148-153) with the diagram referred to as S1-fig. Phase 1 on the diagram refers to FGDs, in text it states ’we designed the questionnaire format and created an initial pool of draft items’. Could this be made clearer for the reader on the diagram please.

Lines 207-210. Please clarify if the ‘study team’ were the same as the ‘study enumerator team’ as described earlier. Do they include the authors? I ask because the FGDs were conducted in Bengali, no mention was made of translating. Were the audio recordings transcribed? It is not clear to me whether the analysis was done immediately as per lines 209 – 212 and as per the debrief.

Line 231 – why 21 cognitive interviews? Why 2-6 interviews per round? Were they conducted in Bengali? Were the interviews transcribed / translated given that they were audio recorded? I realise that the paper contains much detail but this did leave me wondering.

Line 252 – why 13 girls? How selected? Why 2 rounds?
